# Evaluating a Global Clinical Nutrition Program for Pediatric Oncology in Resource-Limited Settings

**DOI:** 10.5334/aogh.5194

**Published:** 2026-07-23

**Authors:** Sofia Leonardo, Federico Antillón, Mulugeta Ayalew, Maria de los Angeles Carrillo, Darrell Manuel Espinoza, Ram Hari Chapagain, Erika Damasco-Avila, Tigist Dawit, Thais Amelia Domínguez, Juna Dhungana, Samantha Ysabelle Ecleo, Sima Ferman, Ligia Fu, Pascale Gassant, Daniel Hailu, Tadele Hailu, Anna Henry, Joyce Kambugu, Francine Kouya, Esther Majaliwa, Barbara Muliro, Mariana Murra, Emanuel Nkya, Irene Nzamu, Wilson Oliveira, Marciel Pedro, Sudhir Sapkota, Trish Scanlan, Joseph Mary Semujju, Carolina Fernandes de Macedo Soares, Tombuh Happiness Sui, Karina Viani, Michelle Walters, Ephrata Yemane, Tsigereda Yitbarek, Stephany Zelaya Sagastizado, Elena J. Ladas

**Affiliations:** 1 Unidad Nacional de Oncología Pediátrica and the School of Medicine, Universidad Francisco Marroquín, Guatemala City, Guatemala; 2 Department of Pediatric Oncology, Gondar University, Gondar, Ethiopia; 3 Department of Pediatric Oncology, Children’s Hospital Manuel de Jesus Rivera, Managua, Nicaragua; 4 Department of Pediatrics, Kanti Children’s Hospital, Kathmandu, Nepal; 5 Division of Hematology, Oncology and Stem Cell Transplantation, Department of Pediatrics, Columbia University Irving Medical Center, New York, USA; 6 St. Damien’s Pediatric Hospital, Port-au-Prince, Haiti; 7 Oncology Unit, Kanti Children’s Hospital, Kathmandu, Nepal; 8 Department of Pediatric Oncology, Philippine General Hospital, Manila, Philippines; 9 Department of Pediatric Oncology, Instituto Nacional de Câncer, Rio de Janeiro, Brazil; 10 Department of Hematology-Oncology, Hospital Escuela Universitario, Tegucigalpa, Honduras; 11 Department of Pediatrics and Child Health, Addis Ababa University, Addis Ababa, Ethiopia; 12 Department of Pediatric Oncology, St. Paul’s Hospital, Addis Ababa, Ethiopia; 13 Department of Paediatric Oncology, Muhimbili National Hospital, Dar es Salaam, Tanzania; 14 Department of Pediatric Oncology, Uganda Cancer Institute, Kampala, Uganda; 15 Department of Pediatric Oncology, Mbingo Baptist Hospital, Bamenda, Cameroon; 16 Department of Pediatric Oncology, Kilimanjaro Christian Medical Center, Moshi, Tanzania; 17 Department of Pediatric Oncology, Kenyatta National Hospital, Nairobi, Kenya; 18 Hospital de Amor, Barretos, Brazil; 19 School of Medicine, University of São Paulo, Sāo Paulo, Brazil

**Keywords:** program evaluation, LMIC, clinical nutrition, capacity building, childhood cancer

## Abstract

*Background:* Children with cancer in low- and middle-income countries (LMICs) experience disproportionately high mortality. Nutritional care improves treatment tolerance and survival, yet access to trained nutrition professionals remains limited. The International Initiative for Pediatrics and Nutrition (IIPAN) was established to strengthen hospital-based clinical nutrition services through capacity-building, education, and mentorship.

*Objectives:* To evaluate the IIPAN global clinical nutrition capacity-building program across hospitals in LMICs.

*Methods:* A monitoring and evaluation framework was implemented across 16 hospitals in 11 countries (February 2024–June 2025). Monthly indicators captured patient characteristics, nutritional status, delivery of nutrition interventions, and dietary diversity. Nutritional status was classified using body mass index (BMI)-for-age, mid-upper arm circumference (MUAC), and height-for-age z-scores based on World Health Organization standards.

*Findings:* Across all sites, 29,295 clinical visits were reported. Most consultations occurred in inpatient settings (79.9%) and were follow-up assessments (85.1%). Among patients, 52.5% had solid tumors and 39.6% had hematologic malignancies. Based on BMI-for-age, 10.3% were classified with severe acute malnutrition (SAM) and 13.5% with moderate acute malnutrition (MAM). Height-for-age analysis indicated that 27.2% were stunted. Nearly all hospitalized patients (95.8%) received a nutrition assessment within 24 hours of admission, and 96.0% received individualized or group nutrition education. Therapeutic foods or formulas were provided in 42.8% of consultations, while advanced nutrition therapy was administered in 3.5%. The prevalence of acute malnutrition decreased significantly between initial and follow-up assessments (38.9% to 25.2%; *P* < 0.001). In sites with food kitchens, provision of therapeutic nutrition was substantially higher than program-wide for both children consuming ≤50% of estimated needs (87.4% vs 55%) and for children with SAM (99.1% vs 86%).

*Conclusions:* The IIPAN model demonstrates that integrated, hospital-based nutrition care led by trained nutritionists can reduce acute malnutrition and improve access to essential nutrition services in LMICs.

## Introduction

From 2020 to 2050, an estimated 13.7 million new cases of childhood cancer will be diagnosed globally [1]. While five-year childhood cancer survival rates now exceed 80% in high-income countries (HICs) [2, 3], survival rates remain as low as 20% in some low- and middle-income countries (LMICs) [4]. Multiple factors contribute to global inequalities in cancer outcomes, including the high prevalence of malnutrition and a lack of supportive care programs [1]. Considering this imbalance in outcomes, nutritional care remains a critical but often underrecognized determinant of outcomes during treatment for childhood cancer by increasing treatment-related toxicities, reducing survival and impacting quality of life [5].

Malnutrition is highly prevalent among children undergoing cancer treatment in LMICs, with reported rates reaching up to 88% in Africa, 66% in Asia, and 79% in Latin America [6]. Patterns differ by diagnosis, with higher burdens of undernutrition reported among children with aggressive solid tumors and significant tumor burden at diagnosis [7, 8], reflecting inherent challenges in accessing timely care in some LMICs. The provision of nutrition care not only reduces complications but is also cost-saving, decreasing per-patient hospitalization costs by up to 36% and contributing to substantial national savings [9, 10]. Yet, these benefits remain largely unrealized in many LMICs. Despite clear evidence supporting individualized nutrition assessment, targeted dietary strategies, and timely therapeutic interventions [5, 11, 12], the ability to implement these practices remains inadequate due to limited personnel, few opportunities for training in clinical dietetics, as well as health system constraints [13].

The International Initiative for Pediatrics and Nutrition (IIPAN) at Columbia University Irving Medical Center operates a global program dedicated to building clinical nutrition capacity and advancing high-quality nutrition research in LMICs, thereby addressing this gap in healthcare endemic to institutions in LMICs [14]. In this report, we detail the impact of our clinical program on the delivery of nutrition care and its associated outcomes in children with cancer.

## Methods

### The IIPAN model

IIPAN is a clinical care and research nutrition program designed to improve nutrition services for children with cancer and other chronic conditions in LMICs. IIPAN supports hospitals through hiring local nutritionists, standardizing clinical training and provision of nutrition services, establishing research standards, and implementing harmonized monitoring systems across participating sites.

IIPAN educates and mentors through a hybrid longitudinal training program. Training is most often launched through an on-site clinical education visit led by a designated IIPAN trainer where hands-on training in pediatric nutrition screening, assessment, intervention, and monitoring in the inpatient and outpatient settings is provided. Programmatic standards-of-practice are established, including medical charting, referral patterns, and collaboration with other departments or programs within the institutions (e.g., nutrition rehabilitation units, hospital dietetics, administration, etc.) that are tailored to each hospital’s resources and guided by the IIPAN manuals [15] to ensure consistency in care across IIPAN sites. Ongoing mentorship is provided via weekly virtual case-discussion calls, creating a supportive environment aimed at improving patient care and strengthening clinicians’ clinical competency. Each session brings together representatives from multiple hospitals, promoting peer-to-peer learning, shared problem-solving, and leadership development. Annual on-site audits are conducted to monitor progress and provide additional support as needed.

IIPAN promotes the use of locally available foods and facilitates partnerships to build capacity for procuring nutrition supplies, therapeutic foods, and formulas. IIPAN’s Food as Medicine program, which operates in Uganda, Ethiopia, and Tanzania, supports the renovation or construction of small hospital kitchens, equipping them with the tools and infrastructure to prepare therapeutic foods and formulas that supplement children’s diets during their stay in the hospital.

### Program monitoring

**Figure 1:**
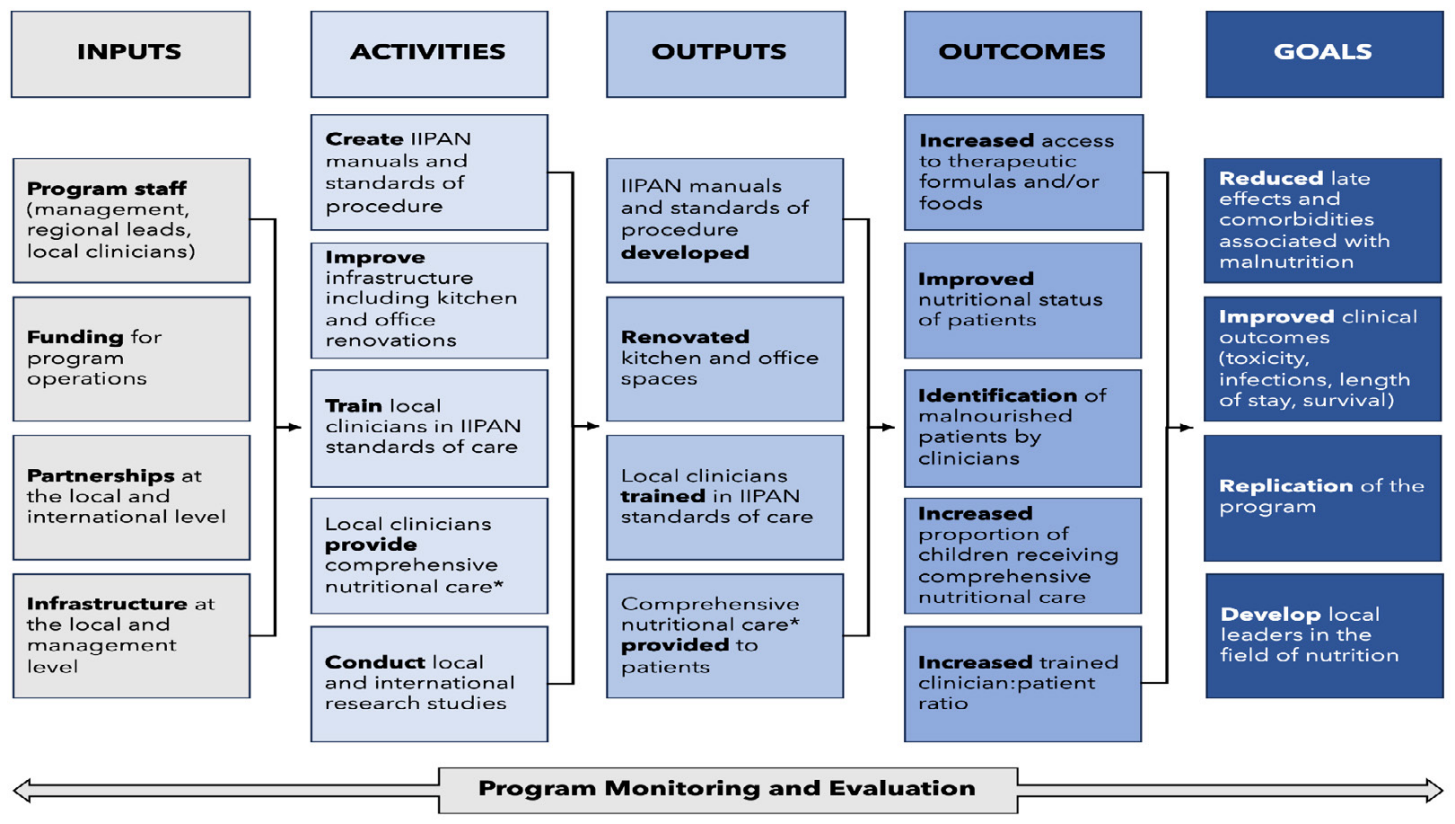
IIPAN Logic model for program monitoring and evaluation.

IIPAN’s logic model defines the program’s intended activities, outputs, and outcomes and establishes indicators to measure progress at each stage (Figure 1) [14]. For this assessment, program indicators were collected across 16 IIPAN affiliated hospitals representing Africa, Asia, Central America and the Caribbean, and South America between February 2024 and June 2025 (Supplementary Figure S1). Program indicators included population characteristics, nutritional status at consultation, delivery of nutrition interventions (e.g., nutrition counseling, provision of therapeutic foods or formulas, and advanced nutrition therapy), and service delivery productivity (e.g., number of clinical nutrition consultations and follow-up assessments). Nutritionists collected program indicators for one week each month. Data were entered into REDCap (Research Electronic Data Capture), a secure, web-based platform hosted at Columbia University Irving Medical Center.

### Programmatic indicators

#### Population characteristics

These included indicators that captured the volume and distribution of nutrition services delivered across sites. Nutritionists documented the total number of consultations, visit type (initial vs follow-up), clinical setting (inpatient vs outpatient), and primary diagnosis. Although the program primarily focuses on children with cancer, nutritionists also provided care to children with other acute or chronic conditions when referred for nutrition assessment as part of routine clinical services. Two hospitals, Saint Damien Pediatric Hospital (Port-au-Prince, Haiti) and Kanti Children’s Hospital (Kathmandu, Nepal), operate hospital-wide nutrition programs.

#### Nutritional status

Nutritional status was assessed using World Health Organization (WHO) growth standards, including body mass index (BMI)-for-age, height-for-age, and mid-upper arm circumference (MUAC). Height-for-age z-scores <−2 and <−3 were classified as stunting and severe stunting, respectively. BMI-for-age z-scores <−2 and <−3 defined moderate acute malnutrition (MAM) and severe acute malnutrition (SAM), respectively. Overweight and obesity were defined using WHO BMI-for-age cutoffs, with age-specific thresholds applied according to WHO methodology [16].

For children with solid tumors, nutritional status was assessed using MUAC due to the often large burden of tumor typically observed at diagnosis in LMICs. For children 6–59 months, <11.5 cm indicated SAM, ≥11.5 and <12.5 cm indicated MAM, and ≥12.5 cm indicated no malnutrition. For children 5–9 years, <13 cm indicated SAM, ≥13.0 and <14.5 cm indicated MAM, and a value ≥14.5 cm indicated no malnutrition. For children 10–15 years, <16 cm indicated SAM, ≥16.0 and <18.5 cm indicated MAM, and ≥18.5 cm indicated no malnutrition [17].

#### Assessment of program impact on nutritional status

To evaluate the program’s impact on nutritional status, follow-up assessments conducted ≥14 days after the initial visit were included. Changes in the prevalence of SAM, MAM, and overweight or obesity were examined at the regional and country levels. Children receiving palliative care were analyzed separately, as their nutritional goals and trajectories differ from those of children receiving active treatment [18].

#### Time to assessment and delivery of nutrition interventions

Nutritionists documented the time from hospital admission to initial evaluation (<24 hours, 24–48 hours, 48–72 hours, or ≥72 hours) to the time to initial consultation. Documentation included individualized nutrition counseling and group education sessions, including the number of sessions, total instructional hours, and number of attendees. Indicators of medical nutrition therapy included administration of therapeutic foods or specialized formulas such as ready-to-use therapeutic foods or high-energy/protein supplements; the proportion of children receiving these products was reported among all children who received therapeutic nutrition, among those with SAM, and among children consuming <50% of estimated energy or protein requirements. Advanced medical nutrition therapy was also documented, including initiation of enteral (EN) or parenteral nutrition (PN) and whether children requiring EN/PN successfully received it.

#### Diet diversity

Minimum dietary diversity was assessed using the Food and Agriculture Organization (FAO)’s Minimum Dietary Diversity for Women (MDD-W) indicator [19], which has been validated for use in children and adolescents in LMICs [20, 21]. For children who were fasting for a medical procedure during the 24 hours preceding the assessment, the last 24-hour period in which the child consumed food was used for dietary recall. The MDD-W is based on the consumption of 10 defined food groups over the previous 24 hours: (1) grains, white roots and tubers, and plantains; (2) pulses; (3) nuts and seeds; (4) dairy; (5) meat, poultry, and fish; (6) eggs; (7) dark green leafy vegetables; (8) other vitamin A–rich fruits and vegetables; (9) other vegetables; and (10) other [19]. For children aged ≥5 years, a score of five or more food groups indicated that MDD was met [21]. For children aged 24–59 months, a cutoff of four or more food groups was used to indicate MDD, as this threshold has been shown to provide better sensitivity, specificity, and correct classification in this age group [20].

#### Program service delivery

Program service delivery indicators assessed the volume of nutrition services delivered across participating sites. Measures included the annual number of clinical and research-related consultations, the average number of follow-up visits per patient, the total hours of group education delivered, the number of education attendees, and the estimated nutritionist-to-patient ratio.

## Statistics

We used descriptive statistics to summarize nutritional status, service delivery indicators, and program population. Categorical variables (e.g., nutritional status categories, receipt of nutrition interventions, and educational activities) were summarized using frequencies and percentages, and continuous variables (e.g., age) were summarized using means and standard deviations. Estimates were stratified by geographic region (Africa, Asia, Central America, and South America) and further disaggregated by country to reflect local variability in program implementation.

Program indicators were summarized using descriptive analyses to characterize implementation of the IIPAN model across sites. Inferential statistical testing was applied only where the data structure supported meaningful comparison, specifically for chi-square tests comparing the prevalence of acute malnutrition and overweight/obesity between initial and follow-up assessments. Sensitivity analyses examining therapeutic nutrition provision at food-kitchen sites were conducted descriptively. All analyses were performed using Stata (StataCorp, College Station, TX, USA), and a p-value <0.05 was considered statistically significant.

## Results

### Program population

The characteristics of children and adolescents receiving care by the IIPAN nutritionist are presented in Table 1, with country-level data provided in Supplementary Table S1. The mean age of patients was 6.6 ± 4.9 years, and 57% were male. Most encounters occurred in inpatient settings (79.9%), and the majority were follow-up nutrition assessments (85.1%). Among patients, 52.5% had solid tumors, 39.6% had hematologic malignancies. Children with cancer (92.1%) represented the majority of patients served by IIPAN followed by children with an acute or chronic condition (7.9%) referred for nutrition care.

**Table 1:** Characteristics of patients by region.

	ALL SITES	AFRICA	ASIA	CENTRAL AMERICA	SOUTH AMERICA
n (%)					
Age (mean ± SD)	6.6 ± 4.9	6.7 ± 4.8	4.9 ± 4.3	8.1 ± 4.8	9.6 ± 5.4
**Sex**					
Male	7,435 (57.0)	5,265 (57.4)	926 (60.6)	888 (51.5)	356 (57.9)
Female	5,607 (43.0)	3,908 (42.6)	603 (39.4)	837 (48.5)	259 (42.1)
**Setting**					
Outpatient	2,438 (20.1)	1,144 (13.4)	281 (19.4)	549 (40.5)	464 (82.5)
Inpatient	9,323 (79.9)	7,364 (86.6)	1,066 (80.6)	797 (59.5)	96 (17.5)
**Type of visit**					
Initial nutrition assessment	1,703 (14.9)	1,274 (15.0)	219 (17.3)	128 (9.9)	82 (20.1)
Follow-up nutrition assessment	9,746 (85.1)	7,366 (85.0)	1,066 (82.7)	1,168 (90.1)	326 (79.9)
**Primary diagnosis**					
Oncologic					
Solid tumor	6,826 (52.5)	5,293 (57.8)	354 (23.5)	671 (39.1)	508 (82.9)
Hematologic	5,153 (39.6)	3,560 (38.8)	653 (43.4)	851 (49.6)	89 (14.5)
Other	1,022 (7.9)	316 (3.4)	498 (33.1)	192 (11.2)	16 (2.6)

Abbreviation: n, number.

### Programmatic indicators of nutritional status

Nutritional status indicators at the time of consultation are summarized by region in Table 2, with corresponding country-level data provided in Supplementary Table S2. Most consultations involved children with a healthy BMI-for-age at initial assessment, undernutrition remained a frequent challenge for nutritionists across IIPAN regions. SAM and MAM were most commonly encountered among children in Asia (30.1%) and Africa (24.7%), whereas Central and South America reported a higher proportion of consultations involving overweight and obesity (9.9% and 30.7%, respectively).

**Table 2:** Nutritional status of patients by region.

	ALL SITES	AFRICA	ASIA	CENTRAL AMERICA	SOUTH AMERICA
n (%)					
**Hematological malignancy or other diagnosis**					
**Nutritional status classified by BMI-for-age z-score**					
SAM	444 (10.3)	307 (10.5)	115 (17.0)	21 (3.2)	1 (2.6)
MAM	578 (13.5)	414 (14.2)	89 (13.1)	74 (11.4)	1 (2.6)
Healthy weight	2,804 (65.4)	1,956 (66.8)	385 (56.8)	440 (68.0)	23 (59.0)
Risk of overweight	146 (3.4)	62 (2.1)	34 (5.0)	48 (7.4)	2 (5.1)
Overweight	215 (5.0)	122 (4.2)	35 (5.2)	48 (7.4)	10 (25.6)
Obese	86 (2.0)	57 (1.9)	14 (2.1)	13 (2.0)	2 (5.1)
Severely obese	17 (0.4)	8 (0.3)	6 (0.9)	3 (0.5)	0 (0.0)
**Solid tumors**					
**Nutritional status classified by MUAC**					
SAM	735 (13.3)	672 (15.0)	17 (5.6)	21 (5.5)	25 (7.1)
MAM	1,036 (18.6)	872 (19.5)	35 (11.5)	75 (19.6)	54 (15.4)
Healthy weight	3,747 (67.9)	2,937 (65.5)	252 (82.9)	286 (74.9)	272 (77.5)
**All diagnoses**					
**Nutritional status classified by height-for-age z-score**					
Severely stunted	1,229 (10.2)	1,049 (12.5)	132 (7.7)	32 (1.9)	16 (4.4)
Stunted	2,063 (17.0)	1,503 (17.9)	316 (18.5)	204 (12.4)	40 (11.0)
Healthy height	8,812 (72.8)	5,827 (69.5)	1,262 (73.8)	1,414 (85.7)	309 (84.7)

Abbreviations: n, number; SAM, severe acute malnutrition; MAM, moderate acute malnutrition; BMI, body mass index; MUAC, mid-upper arm circumference.

Undernutrition was also frequently observed among children with solid tumors when assessed using MUAC (13.3% SAM and 18.6% MAM), particularly in African regions (34.5%). Indicators of chronic undernutrition further reflected long-standing nutritional vulnerability among children receiving care, with 27.2% classified as stunted, including 10.2% with severe stunting. The highest prevalence of stunting and severe stunting was reported in Africa (30.4%) and Asia (26.2%).

### Impact of the IIPAN model on nutritional care

A significant improvement in acute malnutrition was observed across all IIPAN sites after receiving care from an IIPAN nutritionist (Figure 2A). The proportion of children with acute malnutrition decreased from 38.9% to 25.2% between initial and follow-up consultations (≥14 days apart), corresponding to a 13.7% reduction that was statistically significant (*P* < 0.001). The largest reductions were observed in Asia (23.9%; *P* < 0.001) and Central America (17.4%; *P* < 0.001), with smaller but significant improvements in Africa (4.3%; *P* = 0.02). Changes in nutritional status by country are summarized in Supplementary Table S3.

**Figure 2A:**
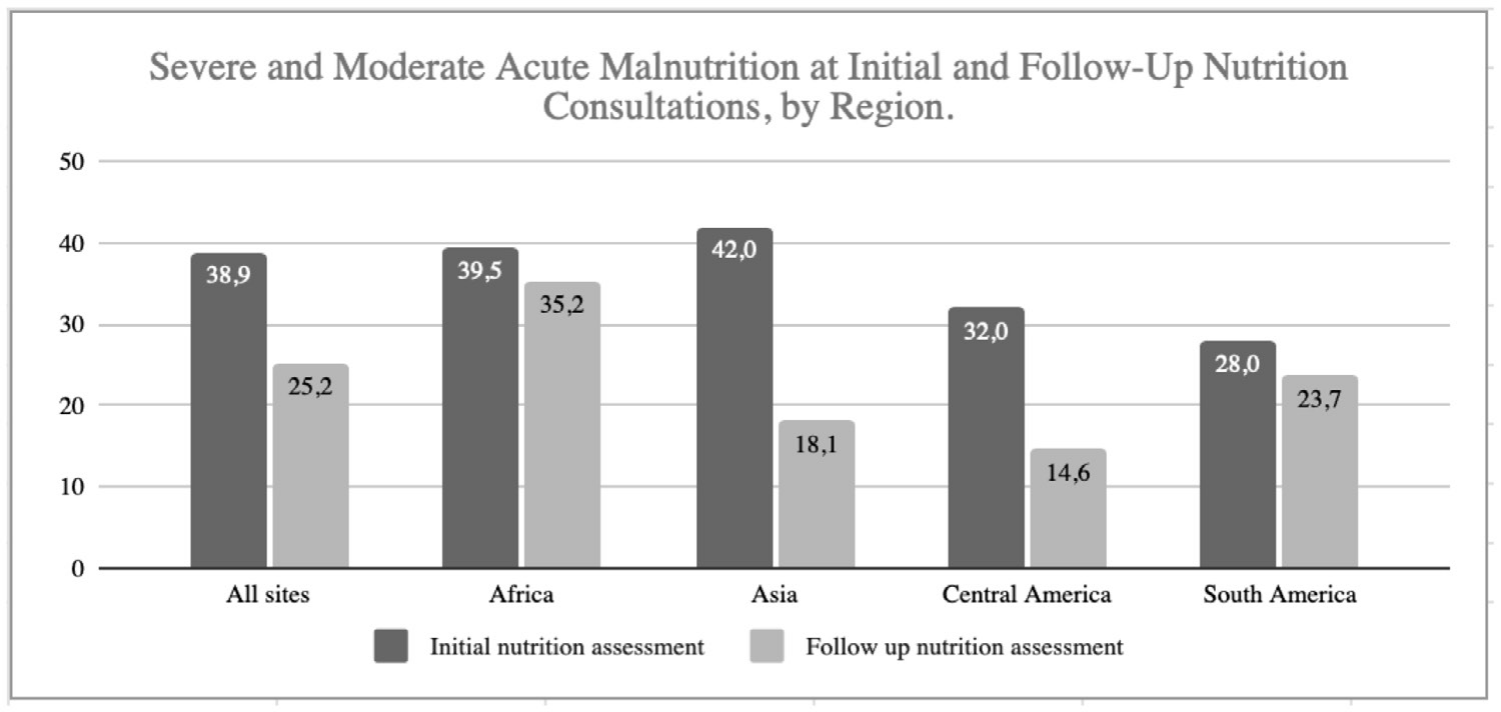
Severe and moderate acute malnutrition at initial and follow-up nutrition consultations, by region. Bars represent the proportion of nutrition consultations classified as moderate or severe acute malnutrition based on body mass index-for-age or mid-upper arm circumference, shown at initial assessment and at follow-up (≥14 days).

In contrast, the proportion of children with overweight or obesity remained relatively stable (Figure 2B). Although there was a small but statistically significant program-wide increase from 10.2% to 12.2% (*P* = 0.02), regional patterns showed minimal variation and were not statistically significant: Africa (*P* = 0.54), Asia (*P* = 0.95), Central America (*P* = 0.41), and South America (*P* = 0.49).

**Figure 2B:**
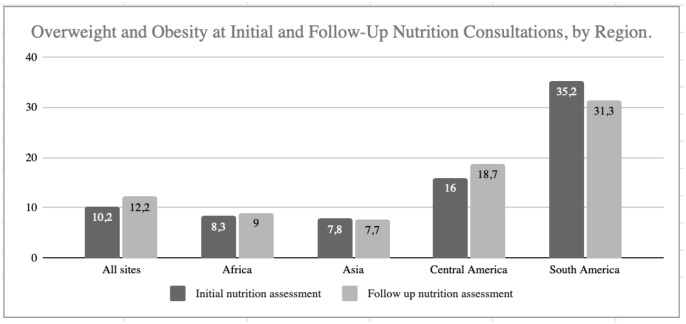
Overweight and obesity at initial and follow-up nutrition consultations, by region. Bars represent the proportion of nutrition consultations classified as overweight or obese based on body mass index-for-age z-scores, shown at initial assessment and at follow-up (≥14 days).

### Time to assessment and delivery of nutrition interventions

Program indicators for nutrition interventions are summarized in Table 3, with country-level data available in Supplementary Table S4. Implementation of IIPAN’s nutrition care model demonstrated timely assessment and provision of core nutrition services across participating regions. Overall, 95.8% of hospitalized children received a nutrition assessment within 24 hours of admission, reflecting strong integration of the IIPAN model into routine clinical care. Similar performance was observed in Africa, South America, and Central America, whereas coverage was lower in Asia (71.4%). The IIPAN model of nutrition counseling was widely integrated into routine service delivery, with 96.0% of consultations including individualized or group education. However, provision of therapeutic foods or formula occurred in only 42.8% of consultations, with the highest distribution observed in Asia (59.5%).

**Table 3:** Time to assessment and delivery of nutrition services by region.

	ALL SITES	AFRICA	ASIA	CENTRAL AMERICA	SOUTH AMERICA
n (%)					
**Time from admission to initial nutrition visit**					
Nutrition assessment within 24 hours of admission	5,133 (95.8)	4,763 (95.8)	5 (71.4)	292 (97.7)	73 (91.3)
Nutrition assessment within 24–48 hours of admission	124 (2.3)	121 (2.4)	0 (0)	3 (1.0)	0 (0)
Nutrition assessment within 48–72 hours of admission	40 (0.7)	35 (0.7)	0 (0)	2 (0.7)	3 (3.8)
Nutrition assessment 72 hours or more after admission	62 (1.2)	54 (1.1)	2 (28.6)	2 (0.7)	4 (5.0)
**Nutrition education**					
Patients receiving individual nutrition education	12,537 (96.0)	8,187 (97.2)	2,491 (97)	1,493 (90.5)	366 (89)
**Therapeutic foods or formula**					
Patients receiving therapeutic foods or formula	5,791 (42.8)	3,993 (47.4)	1,092 (59.5)	540 (32.7)	166 (40.4)
Patients with SAM receiving therapeutic foods or formula	1,695 (86.0)	1,384 (86.6)	211 (82.7)	76 (88.4)	24 (80.0)
Patients meeting 50% or less of calorie or protein needs receiving therapeutic foods or formula	1,052 (55.0)	221 (87.0)	528 (41.8)	278 (74.7)	25 (67.6)
**Enteral or parenteral nutrition**					
Patients receiving enteral or parenteral nutrition	450 (3.5)	227 (2.7)	137 (4.6)	73 (4.4)	13 (3.2)
Patients identified as needing enteral or parenteral nutrition who received it	378 (40.3)	181 (52.6)	135 (29.7)	49 (42.6)	13 (52.0)

Abbreviations: n, number; SAM, severe acute malnutrition.

Among children identified with SAM, 86.0% received therapeutic nutrition, and 55.0% of children meeting ≤50% of energy or protein requirements received supplementation, demonstrating partial but incomplete coverage of identified needs. Advanced nutrition therapy (enteral or parenteral nutrition) was provided in 3.5% of consultations, ranging from 2.7% in Africa to 4.6% in Asia. Due to significant variability in access to industrialized nutrition products among IIPAN institutions, we conducted a sensitivity analysis restricted to the sites with active Food as Medicine kitchens. Combined, these sites provided therapeutic foods or formulas to 87.4% of children consuming ≤50% of their estimated requirements. Similarly, among children with SAM, therapeutic nutrition coverage was 99.1% across IIPAN institutions with Food as Medicine program, compared with 86.0% program-wide.

Fewer than half (40.3%) of children who required advanced nutrition support received it, reflecting significant implementation barriers. Across regions, the most frequently reported challenges included limited availability of supplies, institutional capacity constraints, and parental refusal of enteral feeding, which collectively restricted the provision of advanced therapeutic nutrition.

### Dietary diversity indicator

**Figure 3:**
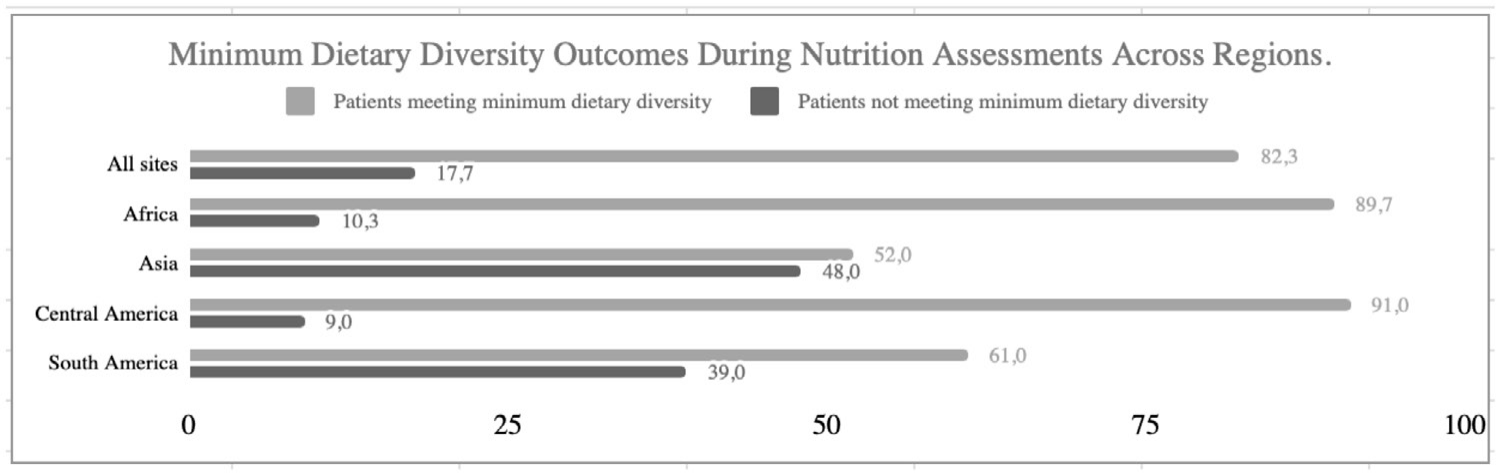
Minimum dietary diversity outcomes during nutrition assessments across regions. Bars represent the proportion of nutrition consultations meeting the Minimum Dietary Diversity threshold based on a 24-hour dietary recall, calculated only for consultations in which dietary recall was performed.

MDD was measured among children who received a 24-hour dietary assessment during nutrition consultations. The proportion of children meeting MDD by region is presented in Figure 3 and by country is presented in Supplementary Table S5. Across participating sites, 82.3% of assessments met the MDD threshold, indicating that nutrition evaluation frequently identified a broad range of food groups consumed within the 24-hour period prior to consultation. The proportion of children meeting MDD was highest in Central America (91.0%) and Africa (89.7%), followed by South America (61.0%), while Asia demonstrated the lowest level (52.0%). However, when MDD was analyzed across IIPAN sites with Food as Medicine programs, the proportions of children meeting the MDD criteria increased to 96.1%, 88.6%, and 85.8% in Tanzania, Ethiopia, and Uganda, respectively.

### Impact of IIPAN model in palliative care

Nutrition indicators for children receiving palliative care are shown in Supplementary Table S6, with country-level results in Supplementary Table S7. Across regions, 379 consultations were reported. Among children with hematologic malignancies or other diagnoses, 47.3% of assessments identified acute malnutrition, most commonly in Asia (60%) and Africa (45.4%). Using MUAC, children with solid tumors showed a higher burden of acute malnutrition (26.8% SAM and 20.7% MAM). In this population, nutritional care was provided within a palliative framework focused on comfort and quality of life rather than nutritional rehabilitation.

### Program service delivery

**Figure 4:**
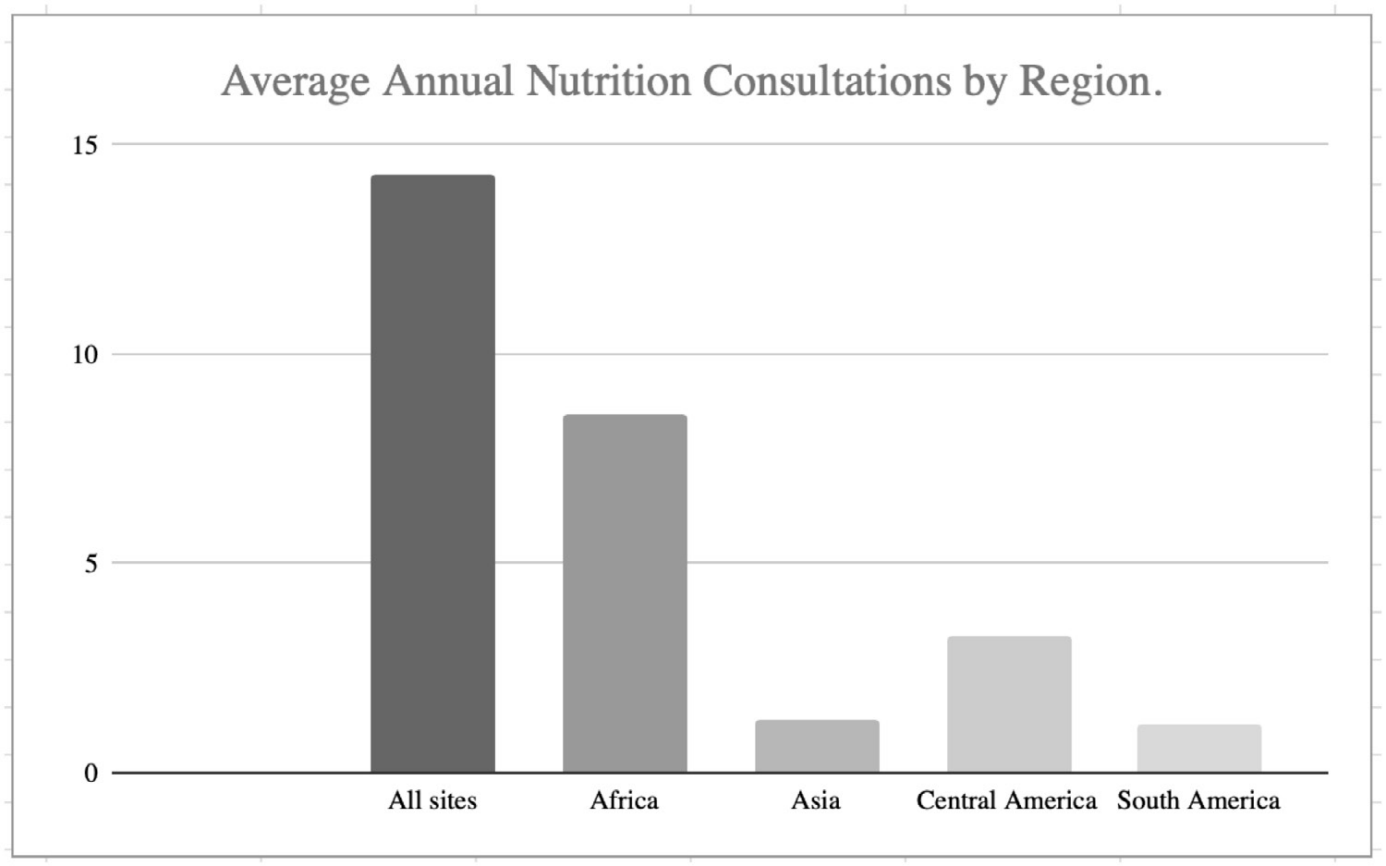
Average annual nutrition consultations by region. Bars represent the total number of nutrition consultations delivered by IIPAN nutritionists across clinical care and research-related activities, shown as annual service volume.

Service delivery productivity indicators are summarized in Table 4, with country-level data in Supplementary Table S8. Across participating regions, nutritionists provided services to an estimated 14,305 nutrition consultations per year, including both clinical care and research-related visits. Service volume was highest in Africa (8,583 consultations), reflecting both the larger number of participating hospitals in this region and the higher patient volume typically managed at these sites. Central America (3,290), Asia (1,287), and South America (1,145) reported lower annual volumes corresponding to fewer participating sites and smaller patient populations (Figure 4).

**Table 4:** Service delivery indicators by region.

	ALL SITES	AFRICA	ASIA	CENTRAL AMERICA	SOUTH AMERICA
n					
Average patients seen per year (clinical + research)	14,331	8,583	1,315	3,288	1,145
Average clinical visits per year	29,295	19,319	2,864	5,955	1,157
Average number of repeat visits per year	2.1	2.4	2.1	1.7	2.8
Hours of group education per year	232	96	2	20	114
Number of attendees receiving education per year	4,355	3,888	140	185	142
Average research visits per year	1,440	245	517	225	453
Nutritionist:patient ratio	1:73	1:64	1:58	1:89	1:116

Abbreviation: n, number.

Across all regions, 29,295 clinical nutrition visits were recorded annually, corresponding to an average of 2.1 documented follow-up assessments per patient per reporting period. In addition, nutritionists delivered 232 hours of group education annually, reaching 4,355 attendees, the majority in Africa (3,888), demonstrating integration of nutrition education within routine care workflows across diverse implementation settings. The estimated nutritionist-to-patient ratio was 1:73, reflecting the average annual number of patients supported per IIPAN nutritionist across participating sites.

## Discussion

Our results illustrate that the IIPAN model, which trains and employs clinical research nutritionists dedicated to caring for children with cancer and other non-communicable diseases, successfully delivers nutritional care in hospitals across Africa, Central and South America, and Asia. Our program offers an economical and scalable approach to bridging critical gaps in nutritional care in resource-limited hospital settings [14]. Each year, IIPAN nutritionists provide individualized care during nearly 30,000 clinical visits and deliver group education to an additional 4,355 individuals, demonstrating its effective reach. Clinical nutrition interventions were delivered in a timely manner across all sites, with nearly 96% of patients receiving an initial nutrition assessment within 24 hours of hospital admission and 96% receiving individualized or group nutrition education. Collectively, these indicators reflect strong integration into routine clinical care models.

We observed a 13.7% reduction in the prevalence of acute malnutrition between initial and follow-up visits spaced at least 14 days apart, highlighting the program’s adaptive guidelines in effectively remediating acute malnutrition [15]. Greater reductions may have been observed with longer follow-up, as nutritional rehabilitation typically requires six weeks to three months [22]. In contrast, the prevalence of overweight and obesity remained relatively stable between visits. This finding is consistent with evidence indicating that management of excess weight in children and adolescents often requires at least 26 contact hours [23].

Across Africa, Asia, and Central America, malnutrition among children with cancer was consistently lower at IIPAN-supported hospitals than previously reported, suggesting a meaningful impact of IIPAN’s nutritional interventions. In Africa, malnutrition prevalence among the IIPAN sites was 24.7% by BMI-for-age and 34.5% by MUAC, compared with published rates as high as 67.6% in Ethiopia and 41.5% in Tanzania [24, 25]. In Central America, where undernutrition prevalence has been reported as high as 63%, IIPAN-supported hospitals reported a substantially lower rate of 25.1% [26]. Similarly, in Nepal, previously reported undernutrition rates of 44% among children with acute lymphoblastic leukemia (ALL) exceeded the 33.9% observed in our program [27]. At the outpatient site in Brazil, the prevalence of acute malnutrition (22.5%) and overweight or obesity (30.7%) fell within published ranges [28]. Overall, these comparisons indicate that the IIPAN model is effective at reducing undernutrition in pediatric oncology wards among different regions in the globe.

We observed regional variation in nutritional patterns, with SAM and MAM most frequently identified in Asia and Africa, while overweight and obesity were more common in Central and South America. These patterns are consistent with national and regional reports describing a high burden of undernutrition in South Asia and sub-Saharan Africa and a rising prevalence of childhood overweight and obesity in Latin America [29, 30]. This variation underscores the need to adapt clinical nutrition strategies to regional contexts, as a single approach is insufficient to address the diverse nutritional challenges across a global program. Accordingly, training in Central and South America emphasizes the management of overweight and obesity and the implications of excess weight in childhood cancer, with IIPAN virtual training calls organized regionally to align with these differing challenges and local contextual factors.

The global prevalence of SAM and MAM among children with palliative care was 47.3% higher than the overall rates observed among all patients seen by IIPAN nutritionists. In the context of palliative care, IIPAN’s nutritional approach prioritizes quality of life rather than restoration of nutritional status. Nutritional counseling for children receiving palliative care focuses on the emotional, social, and comforting aspects of eating and drinking, rather than on meeting full caloric or protein requirements. This is consistent with nutritional care principles for patients in palliative care, which recognize that appetite and nutritional needs naturally decline at the end of life, and that more aggressive nutritional interventions may not align with patient comfort or family goals [18].

Our model also highlights persistent health system limitations in delivering optimal nutrition care. Fewer than half of patients seen by an IIPAN nutritionist received therapeutic foods or formula, and only 55.0% of those meeting ≤50% of their caloric or protein needs received supplementation. Although IIPAN nutritionists prioritized therapeutic nutrition for children with SAM, of whom 86.0% received therapeutic foods or formula (i.e., F100), the overall distribution of nutrition supplements remained limited, likely impacting the true impact of the IIPAN model in clinical care. Ready-to-use therapeutic food (RUTF), the standard treatment for SAM, can cost US$41–51 per child over the course of treatment, limiting sustainability and accessibility in many settings [31]. In low-resource hospitals, therapeutic foods and formulas are often out of stock, intermittently available through donations, or restricted by strict eligibility criteria. Similar barriers have been reported across integrated nutrition programs within health systems [32].

It is also important to note that RUTF and F100 are not designed to provide ongoing support for children with chronic diseases. Thus, our model proposes alternative solutions to optimize nutritional care for hospitalized children. The Food as Medicine program is a scalable, cost-effective approach that equips hospital kitchens to prepare nutrient-dense, context-appropriate foods as alternatives to commercial therapeutic products. Building on longstanding practices of locally enriched family foods [33], sites implementing Food as Medicine demonstrated stronger delivery of therapeutic nutrition. Collectively, these findings underscore the importance of locally driven, sustainable models to reduce barriers to nutrition care in LMIC.

While initiatives such as local food kitchens address many nutritional needs, advanced interventions, including enteral and parenteral nutrition, remain largely inaccessible in LMIC settings due to limited supplies and the need for specialized resources [34]. As a result, only 3.5% of patients across IIPAN sites received advanced nutrition therapy, and just 40.3% of those identified as requiring such interventions received them. Persistent barriers, including resource constraints and parental refusal of enteral feeding, have been widely reported [35, 36], and remain challenges that IIPAN seeks to address through partnerships and peer-to-peer education. Beyond therapeutic interventions, IIPAN emphasizes dietary quality and diversity through nutrition education and Food as Medicine programs, which may contribute to higher-than-expected dietary diversity among participating children [37, 38]. However, differences in MDD were observed across regions, with higher proportions of children meeting MDD criteria in Africa and Central America than in Asia and South America. These variations in standards of practice should be interpreted in the context of regional and site-level heterogeneity in food systems, dietary practices, clinical setting, and availability of hospital-based nutrition support. For example, sites with local food kitchens may have greater capacity to provide prepared meals or supplements that include multiple food groups during hospitalization. Similarly, differences in inpatient versus outpatient service delivery may influence the extent to which dietary intake reflects hospital-provided foods, home diets, or family food access. These findings reinforce the value of IIPAN’s adaptable clinical model, which allows nutrition education and food-based programs to be tailored to local food environments, institutional resources, and care models.

Across all IIPAN sites, there was one nutritionist for every 73 patients, with ratios ranging from 1:43 in Cameroon to 1:134 in Nicaragua. Although no single benchmark exists for nutrition staffing in resource-limited pediatric oncology settings, available reference points vary widely, from intensive models in the United States to substantially larger patient loads reported elsewhere [39–41]. Within this context, IIPAN staffing levels support measurable improvements in nutritional status, often through having a nutritionist dedicated exclusively to pediatric oncology. Such department-dedicated roles remain uncommon in many LMIC hospitals, underscoring the importance of optimizing nutrition workforce capacity to deliver effective care [42–44].

In conclusion, the IIPAN model demonstrates that integrated, hospital-based nutrition programs led by trained nutritionists can measurably improve nutritional outcomes among children with cancer and non-communicable diseases in resource-limited settings. Across diverse geographies and clinical contexts, IIPAN’s approach has reduced the burden of acute malnutrition and improved timely access to care. By implementing context-appropriate interventions such as the scalable Food as Medicine program, IIPAN offers a scalable, sustainable model for embedding nutrition into clinical care.

## Data Availability

The data that support the findings of this program evaluation are available from the corresponding author upon reasonable request.

## Additional File

The additional file for this article can be found as follows:
