# Supplementary material for: Evaluating a Global Clinical Nutrition Program for Pediatric Oncology in Resource-Limited Settings
Source: Ann Glob Health. 2026 Jul 23;92(1):72. doi: 10.5334/aogh.5194 (PMC13426452; doi:10.5334/aogh.5194)
Supplement: Supplementary material. — Figure S1 and Tables S1 to S8. [file agh-92-1-5194-s1.pdf]

Supplementary Figure 1. Geographic Distribution of IIPAN Program Sites

The International Initiative for Pediatrics and Nutrition's Global Reach

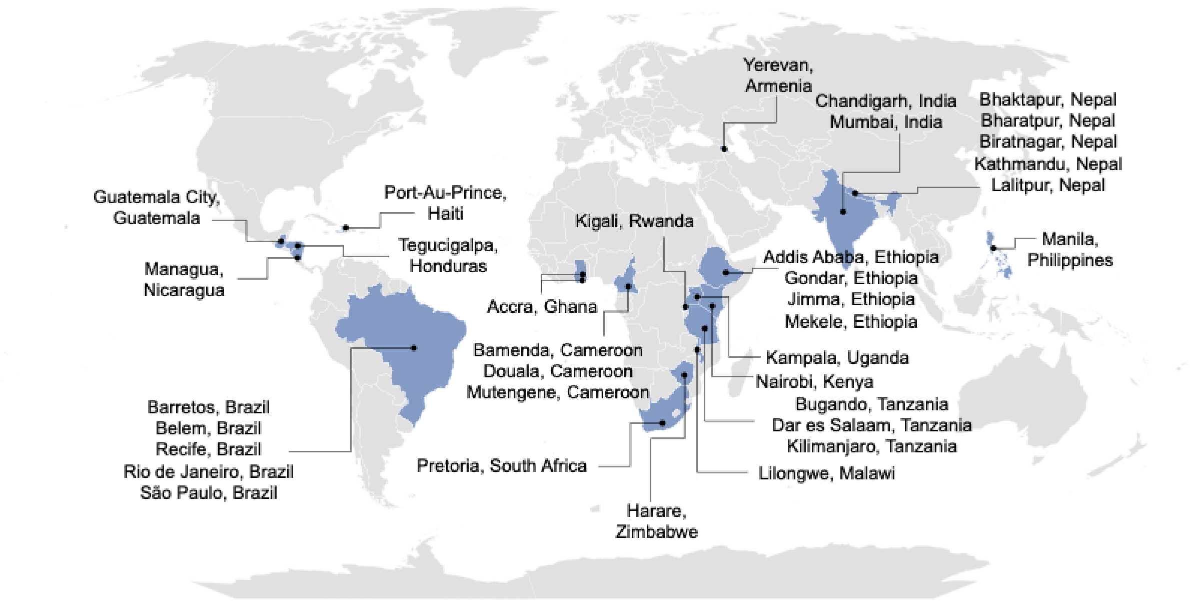

Hospitals Affiliated with the International Initiative for Pediatrics and Nutrition Collecting Program Indicators

Africa:
Gondar University Specialized Hospital (Gondar, Ethiopia)
Kenyatta National Hospital (Nairobi, Kenya)
Kilimanjaro Christian Medical Center (Kilimanjaro, Tanzania)
Mbingo Baptist Hospital (Bamenda, Cameroon)
Muhimbili National Hospital (Dar es Salaam, Tanzania)
St. Paul's Hospital (Addis Ababa, Ethiopia)
Tikur Anbessa Specialized Hospital (Addis Ababa, Ethiopia)
Uganda Cancer Institute (Kampala, Uganda)

Asia:
Kanti Children's Hospital (Kathmandu, Nepal)
Philippine General Hospital (Manila, Philippines)

Central America & the Carribean:
Hospital Escuela (Tegucigalpa, Honduras)
Manuel de Jesús Rivera "La Mascota" Children's Hospital (Managua, Nicaragua)
Saint Damien Pediatric Hospital (Port-Au-Prince, Haiti)
Unidad Nacional de Oncología Pediátrica (Guatemala City, Guatemala)

South America:
Hospital de Amor Infantojuvenil (Barretos, Brazil)
Instituto Nacional de Câncer (Rio de Janeiro, Brazil)

Supplementary Table 1. Characteristics of Patients by Country

Characteristics	Kenya	Tanzania	Uganda	Ethiopia	Cameroon	Brazil	Honduras	Guatemala	Nicaragua	Haiti	Nepal	Philippines
	n (%)											
Age (mean ± SD)	6.2 ± 3.6	6.4 ± 4.9	9.9 ± 5.1	6.2 ± 4.0	9.5 ± 4.1	9.6 ± 5.4	9.0 ± 5.1	11.0 ± 5.0	6.8 ± 3.5	7.3 ± 4.9	4.5 ± 3.9	8.8 ± 5.2
**Sex**												
Male	1,111 (57.3)	2,964 (56.2)	567 (59.7)	356 (63.6)	267 (58.81)	356 (57.9)	278 (48.4)	23 (67.6)	270 (49.1)	317 (55.9)	753 (60.2)	173 (62.2)
Female	828 (42.7)	2,307 (43.8)	382 (40.3)	204 (36.4)	187 (41.19)	259 (41.1)	296 (51.6)	11 (32.4)	280 (50.9)	250 (44.1)	498 (39.8)	105 (37.8)
**Setting**												
Outpatient	228 (13.8)	403 (8.1)	0 (0.0)	120 (22.5)	393 (77.2)	464 (82.9)	197 (39.1)	30 (85.7)	196 (45.0)	126 (34.0)	243 (21.8)	38 (16.5)
Inpatient	1,424 (86.2)	4,576 (91.9)	836 (100)	414 (77.5)	116 (22.8)	96 (17.1)	307 (60.9)	5 (14.3)	240 (55.0)	245 (66.0)	873 (78.2)	193 (83.5)
**Type of visit**												
Initial	163 (9.9)	398 (8.0)	379 (45.3)	246 (46.1)	88 (17.4)	82 (20.1)	15 (3.1)	-	44 (10.1)	69 (18.6)	191 (18.5)	28 (12.1)
Follow up	1,489 (90.1)	4,555 (92.0)	457 (54.7)	288 (53.9)	417 (82.6)	326 (79.9)	474 (96.9)	-	392 (89.9)	302 (81.4)	843 (81.5)	203 (87.9)
**Primary Diagnosis**												
Oncologic												
Solid tumor	1,085 (56.0)	3,135 (59.5)	606 (63.9)	262 (46.5)	205 (45.4)	508 (82.9)	172 (30.6)	0 (0.0)	264 (48.0)	235 (41.4)	200 (12.3)	154 (55.6)
Hematologic	849 (43.8)	1,946 (37.0)	227 (23.9)	292 (51.8)	246 (54.4)	89 (14.5)	383 (68.0)	34 (100.0)	286 (52.0)	148 (26.1)	544 (44.3)	109 (39.4)
Other	5 (0.3)	184 (3.5)	116 (12.2)	10 (1.8)	1 (0.2)	16 (2.6)	8 (1.4)	0 (0.0)	0 (0.0)	184 (32.5)	484 (43.4)	14 (5.1)

Abbreviation: n, number.

Supplementary Table 2. **Nutritional Status of Patients by Country**

	Kenya	Tanzania	Uganda	Ethiopia	Cameroon	Brazil	Honduras	Nicaragua	Haiti	Nepal	Philippines
n (%)											
**Hematological malignancy or other diagnosis**											
**Nutritional status classified by BMI-for-age z-score**											
SAM	15 (2.5)	206 (11.5)	18 (10.8)	68 (27.5)	0 (0.0)	1 (2.6)	7 (2.5)	0 (0.0)	14 (8.2)	111 (19.4)	4 (3.8)
MAM	45 (7.6)	275 (15.3)	26 (15.6)	53 (21.5)	15 (12.4)	1 (2.6)	23 (8.3)	19 (9.6)	32 (18.7)	83 (14.5)	6 (5.7)
Healthy weight	429 (72.1)	1,207 (67.2)	115 (68.9)	111 (44.9)	94 (77.7)	23 (59.0)	193 (69.4)	139 (70.2)	108 (63.2)	321 (56.0)	64 (61.0)
Risk of overweight	31 (5.2)	21 (1.2)	1 (0.6)	9 (3.6)	0 (0.0)	2 (5.1)	23 (8.3)	13 (6.6)	12 (7.0)	24 (4.2)	10 (9.5)
Overweight	63 (10.6)	39 (2.2)	6 (3.6)	5 (2.0)	9 (7.4)	10 (25.6)	31 (11.2)	13 (6.6)	4 (2.3)	25 (4.4)	10 (9.5)
Obese	10 (1.7)	45 (2.5)	0 (0.0)	1 (0.4)	1 (0.8)	2 (5.1)	1 (0.4)	11 (5.6)	1 (0.6)	8 (1.4)	6 (5.7)
Severely obese	2 (0.3)	3 (0.2)	1 (0.6)	0 (0.0)	2 (1.7)	0 (0.0)	0 (0.0)	3 (1.5)	0 (0.0)	1 (0.2)	5 (4.8)
**Solid tumors**											
**Nutritional status classified by MUAC**											
SAM	147 (16.6)	304 (10.9)	129 (33.0)	65 (27.2)	27 (16.1)	25 (7.1)	10 (9.2)	11 (7.6)	0 (0.0)	2 (1.2)	15 (10.9)
MAM	168 (18.9)	551 (19.7)	67 (17.1)	63 (26.4)	23 (13.7)	54 (15.4)	16 (14.7)	45 (31.0)	14 (10.9)	14 (8.4)	21 (15.3)
Healthy weight	573 (64.5)	1,940 (69.4)	195 (49.9)	111 (46.5)	118 (70.2)	272 (77.5)	83 (76.2)	89 (61.4)	114 (89.1)	151 (90.4)	101 (73.7)
**All diagnoses**											
**Nutritional status classified by height-for-age z-score**											
Severely stunted	80 (5.2)	853 (16.5)	21 (3.2)	60 (11.2)	35 (7.2)	16 (4.4)	5 (0.6)	3 (0.7)	24 (6.5)	112 (7.7)	20 (8.0)
Stunted	210 (13.8)	983 (19.0)	123 (19.0)	97 (18.1)	90 (18.4)	40 (11.0)	92 (11.0)	72 (16.5)	40 (10.8)	296 (20.3)	19 (7.6)
Healthy height	1,237 (81.0)	3,342 (64.5)	506 (77.8)	378 (70.7)	364 (74.4)	309 (84.6)	747 (88.4)	361 (82.8)	306 (82.7)	1,052 (72.1)	210 (84.3)

Abbreviations: n, number; SAM, severe acute malnutrition; MAM, moderate acute malnutrition; BMI, body mass index; MUAC, mid-upper arm circumference.

Supplementary Table 3. Prevalence of Malnutrition at Initial and Follow-Up Nutrition Assessments by Country

**Severe and Moderate Acute Malnutrition**											
	**Kenya**	**Tanzania**	**Uganda**	**Ethiopia**	**Cameroon**	**Brazil**	**Honduras**	**Nicaragua**	**Haiti**	**Nepal**	**Philippines**
	n/total n (%)										
**Initial nutrition assessment**	73/228 (32.0)	190/527 (36.1)	187/448 (41.7)	138/308 (44.8)	48/100 (48.0)	35/125 (28)	5/25 (20)	16/61 (26.2)	33/83 (39.8)	175/402 (43.5)	11/39 (28.2)
**Follow up nutrition assessment**	93/386 (24.1)	171/527 (32.5)	75/131 (57.3)	80/107 (74.8)	46/170 (27.1)	41/173 (23.7)	52/348 (14.9)	54/308 (17.5)	3/89 (3.4)	96/582 (16.5)	18/74 (24.3)
**Overweight and Obesity**											
	**Kenya**	**Tanzania**	**Uganda**	**Ethiopia**	**Cameroon**	**Brazil**	**Honduras**	**Nicaragua**	**Haiti**	**Nepal**	**Philippines**
	n/total n (%)										
**Initial nutrition assessment**	32/228 (14.0)	37/527 (7.0)	24/448 (5.4)	36/308 (11.7)	5/100 (5.0)	44/125 (35.2)	5/25 (20.0)	19/61 (31.1)	3/83 (3.6)	25/402 (6.2)	8/19 (42.1)
**Follow up nutrition assessment**	52/358 (14.5)	39/556 (7.0)	4/143 (2.8)	5/76 (6.6)	14/140 (10.0)	45/144 (31.3)	77/369 (20.9)	49/219 (22.4)	5/112 (4.5)	36/612 (5.9)	17/73 (23.3)

Abbreviation: n, number.

Supplementary Table 4. Time to Assessment and Delivery of Nutrition Services by Country

	Kenya	Tanzania	Uganda	Ethiopia	Cameroon	Brazil	Honduras	Nicaragua	Haiti	Nepal	Philippines
n (%)											
**Time from admission to initial nutrition visit**											
Nutrition assessment within 24 hours of admission	445 (98.9)	3,916 (97.8)	282 (80.3)	101 (66.5)	19 (100.0)	73 (91.2)	2 (40)	131 (97.7)	159 (99.4)	2 (100.0)	3 (60.0)
Nutrition assessment within 24-48 hours of admission	5 (1.1)	23 (0.6)	65 (18.5)	28 (18.4)	0 (0.0)	0 (0.0)	1 (20)	1 (0.7)	1 (0.6)	0 (0.0)	0 (0.0)
Nutrition assessment within 48-72 hours of admission	0 (0.0)	25 (0.6)	4 (1.1)	6 (3.9)	0 (0.0)	3 (3.8)	2 (40)	0 (0.0)	0 (0.0)	0 (0.0)	0 (0.0)
Nutrition assessment 72 hours or more after admission	0 (0.0)	37 (0.9)	0 (0.0)	17 (11.2)	0 (0.0)	4 (5.0)	0 (0.0)	2 (1.5)	0 (0.0)	0 (0.0)	2 (40)
**Nutrition education**											
Patients receiving individual nutrition education	1,449 (90.0)	5,067 (100.0)	667 (98.2)	519 (97.2)	485 (96.4)	366 (89.0)	703 (82.8)	421 (98.0)	369 (99.5)	2,298 (99.0)	193 (79.0)
**Therapeutic foods or formula**											
Patients receiving therapeutic foods or formula	293 (18.2)	2,774 (54.2)	436 (64.2)	362 (67.8)	128 (25.5)	166 (40.4)	293 (34.5)	162 (37.6)	85 (23.0)	917 (39.3)	175 (69.0)
Patients with SAM receiving therapeutic foods or formula	136 (61.5)	717 (86.6)	270 (99.3)	195 (95.6)	66 (89.2)	24 (80.0)	39 (90.7)	18 (81.8)	19 (90.5)	178 (82.4)	33 (84.6)
Patients meeting 50% or less of calorie or protein needs receiving therapeutic foods or formula	23 (92.0)	16 (100.0)	103 (87.3)	48 (84.2)	31 (81.6)	25 (67.6)	219 (78.5)	45 (73.8)	14 (63.6)	416 (38.3)	112 (64.0)
**Enteral or parenteral nutrition**											
Patients receiving enteral or parenteral nutrition	43 (2.7)	92 (1.8)	38 (5.6)	49(9.2)	5 (1.0)	13 (3.2)	26 (3.1)	38 (8.7)	9 (2.4)	121 (52.1)	16 (6.5)
Patients identified as needing enteral or parenteral nutrition who received it	39 (47.6)	59 (62.1)	35 (36.0)	45 (80.4)	3 (21.4)	13 (52.0)	20 (38.5)	26 (43.3)	3 (100.0)	119 (31.3)	15 (20.3)

Abbreviations: n, number; SAM, severe acute malnutrition.

Supplementary Table 5. Minimum Dietary Diversity Among Children Receiving Nutritional Care Across Countries

	Kenya	Tanzania	Uganda	Ethiopia	Cameroon	Brazil	Honduras	Nicaragua	Haiti	Nepal	Philippines
n (%)											
**Patients meeting minimum dietary diversity**	108 (87.8)	512 (96.1)	265 (85.8)	31 (88.6)	129 (87.8)	105 (61.0)	25 (61.0)	126 (91.3)	131 (100.0)	118 (52.0)	30 (55.0)
**Patients not meeting minimum dietary diversity**	29 (12.2)	37 (3.9)	44 (14.2)	4 (11.4)	18 (12.3)	67 (39.0)	16 (39.0)	12 (8.7)	0 (0.0)	109 (48.0)	25 (45.0)

Supplementary Table 6. Nutrition Indicators Among Children Receiving Palliative Care by Region

	All sites	Africa	Asia	Central America	South America
	n (%)				
**Hematological malignancy or other diagnosis**					
**Nutritional status classified by BMI-for-age z-score**					
SAM	1 (2.6)	1 (4.5)	0	0	0
MAM	17 (44.7)	9 (40.9)	6 (60)	2 (33.3)	0
Healthy weight	19 (50)	12 (54.5)	3 (30)	4 (66.7)	0
Risk of overweight	0	0	0	0	0
Overweight	1 (2.6)	0	1 (10)	0	0
Obese	0	0	0	0	0
Severely obese	0	0	0	0	0
**Solid tumors**					
**Nutritional status classified by MUAC**					
SAM	53 (26.8)	50 (36.5)	2 (11.1)	1 (2.3)	0
MAM	41 (20.7)	33 (24.1)	1 (5.6)	7 (16.3)	0
Healthy weight	104 (52.5)	54 (39.4)	15 (83.3)	35 (81.4)	0
**All diagnoses**					
**Nutritional status classified by height-for-age z-score**					
Severely stunted	17 (11.9)	15 (19.5)	2 (10.5)	0	0
Stunted	23 (16.1)	15 (19.5)	3 (15.8)	5 (11.1)	0
Healthy height	103 (72)	47 (61)	14 (73.7)	40 (88.9)	2 (100)

Abbreviations: n, number; SAM, severe acute malnutrition; MAM, moderate acute malnutrition; BMI, body mass index; MUAC, mid-upper arm circumference.

Supplementary Table 7. Nutrition Indicators Among Children Receiving Palliative Care by Country

	Kenya	Tanzania	Uganda	Ethiopia	Cameroon	Brazil	Honduras	Nicaragua	Haiti	Nepal	Philippines
n (%)											
**Hematological malignancy or other diagnosis**											
**Nutritional status classified by BMI-for-age z-score**											
SAM	0	0	0	1 (100)	0	0	0	0	0	0	0
MAM	0	4 (80)	0	0	5 (35.7)	0	0	2 (40)	0	1 (25)	5 (83.3)
Healthy weight	0	1 (20)	2 (100)	0	9(64.3)	0	0	3 (60)	1 (100)	2 (50)	1 (16.7)
Risk of overweight	0	0	0	0	0	0	0	0	0	0	0
Overweight	0	0	0	0	0	0	0	0	0	1 (25)	0
Obese	0	0	0	0	0	0	0	0	0	0	0
Severely obese	0	0	0	0	0	0	0	0	0	0	0
**Solid tumors**											
**Nutritional status classified by height-for-age z-score**											
SAM	24 (38.1)	7 (87.5)	11 (42.3)	0	8 (21.6)	0	1 (33.3)	0	0	2 (25)	0
MAM	21 (33.3)	0	6 (23.1)	3 (100)	3 (8.1)	0	2 (66.7)	4 (36.4)	1 (3.4)	1 (12.5)	0
Healthy weight	18 (28.6)	1 (12.5)	9 (34.6)	0	26 (70.3)	0	0	7 (63.6)	28 (96.6)	5 (62.5)	10 (100)
**All diagnoses**											
**Nutritional status classified by height-for-age z-score**											
Severely stunted	5 (20)	1 (12.5)	0	1 (33.3)	8 (25.8)	0	0	0	0	2 (25)	0
Stunted	5 (20)	5 (25)	0	1 (33.3)	4 (12.9)	0	1 (20)	1 (9.1)	3 (10.3)	3 (37.5)	0
Healthy height	15 (60)	5 (62.5)	0	1 (33.3)	26 (61.3)	2 (100)	4 (80)	10 (90.9)	26 (89.7)	3 (37.5)	11 (100)

Abbreviations: n, number; SAM, severe acute malnutrition; MAM, moderate acute malnutrition; BMI, body mass index; MUAC, mid-upper arm circumference.

Supplementary Table 8. Service Delivery Indicators by Country

	Kenya	Tanzania	Uganda	Ethiopia	Cameroon	Brazil	Honduras	Guatemala	Nicaragua	Haiti	Nepal	Philippines
						n						
Average patients seen per year (clinical + research)	2,849	3,074	1,115	716	829	1,145	584	157	1,759	786	972	315
Average clinical visits per year	5,798	7,922	2,799	1,174	1,626	1,157	1,406	NA	3,056	1,493	2,270	594
Average number of repeat visits per year	1.72	4.32	2.76	0.89	3.89	2.34	1.56	0.67	1.78	2.56	1.65	1.46
Hours of group education per year	3.92	50	19.55	18.5	3.92	114	10	NA	2	7.58	0.67	1.75
Number of attendees receiving education per year	274	1,282	1,467	656	209	142	22	NA	18	145	45	95
Average research visits per year	N/A	245	N/A	N/A	N/A	453	77	148	N/A	N/A	517	N/A
Nutritionist:patient ratio	1:87	1:45	1:70	1:60	1:43	1:116	1:101	1:73	1:134	1:31	1:56	1:59

Abbreviations: n, number; N/A, not applicable.
